# 4.P. Workshop: Health promotion policy implementation evaluation: tools, challenges and futures directions

**DOI:** 10.1093/eurpub/ckac129.267

**Published:** 2022-10-25

**Authors:** Organised by: University of Limerick (Ireland), Chair persons: Aurélie Van Hoye (Ireland), Catherine Woods (Ireland)

## Abstract

**Background:**

Public policies can enhance opportunities for the whole populations to be more health promoting. They are important tools as they tackle the upstream component of health promotion behaviours to influence the environment and opportunities for the general population. Research has focused on policy development and content, but less on its implementation process and s evaluation. These latter steps are necessary for policy accountability, would serve policy-making process and strengthen policy impact for real change.

**Objectives:**

The objectives of the workshop are to present different methods and toolkits allowing the evaluation of policy implementation at national level.

**Workshop format:**

The workshop will include 4 presentations, with two systematic reviews and two tool presentation, to offer opportunities to the audience to understand both theoretical and methodological perspective and applied process in regard to health promotion policy evaluation and implementation evaluation. First, a systematic literature review considering the fitting framework for implementation of policies promoting health nutrition and physically active lifestyle will describe the components and scope of 18 policy implementation frameworks. Second, a systematic literature review on methods of physical activity policy monitoring will help us to learn from the analysis of 112 studies on research-driven, government-driven or co-production approaches, their strengths and weaknesses. The third presentation will introduce the Physical Activity Environment Policy Index, a tool to monitor and benchmark policies to improve the healthiness of physical activity policy environment, as well as provide guidance on how to use it. The fourth presentation will present the health promoting sports clubs national audit tool, aimed at reviewing policy supporting sports clubs to promote health, and present preliminary results from its use in Ireland. During the presentation we will pose questions to the audience, encouraging them to apply the theory/tools to their own context, and to spark conversation and interaction as part of the workshop. After the presentations, questions from the audience will be discussed with each presenter, and a discussion will be open in terms of future challenges for health promotion policies implementation evaluation.

**Key messages:**

• Guidance is needed to evaluate health promotion policies implementation to improve practice, as well as development of framework and methods to build toolkits.

• Policy implementation evaluation is key for improving policy making process.

